# A human rights approach to the health implications of food and nutrition insecurity

**DOI:** 10.1186/s40985-017-0056-5

**Published:** 2017-03-09

**Authors:** Ana Ayala, Benjamin Mason Meier

**Affiliations:** 1 O’Neill Institute for National and Global Health Law, Georgetown University Law Center, 600 New Jersey Avenue NW, Washington DC, USA; 2 Global Health Policy at the University of North Carolina at Chapel Hill, Chapel Hill, NC USA

**Keywords:** Food security, Nutrition security, Malnutrition, Human rights, Right to food, Right to health

## Abstract

Food and nutrition insecurity continues to pose a serious global challenge, reflecting government shortcomings in meeting international obligations to ensure the availability, accessibility, and quality of food and to ensure the highest attainable standard of health of their peoples. With global drivers like climate change, urbanization, greater armed conflict, and the globalization of unhealthy diet, particularly in under-resourced countries, food insecurity is rapidly becoming an even greater challenge for those living in poverty. International human rights law can serve a critical role in guiding governments that are struggling to protect the health of their populations, particularly among the most susceptible groups, in responding to food and nutrition insecurity. This article explores and advocates for a human rights approach to food and nutrition security, specifically identifying legal mechanisms to “domesticate” relevant international human rights standards through national policy. Recognizing nutrition security as a determinant of public health, this article recognizes the important links between the four main elements of food security (i.e., availability, stability, utilization, and access) and the normative attributes of the right to health and the right to food (i.e., availability, accessibility, affordability, and quality). In drawing from the evolution of international human rights instruments, official documents issued by international human rights treaty bodies, as well as past scholarship at the intersection of the right to health and right to food, this article interprets and articulates the intersectional rights-based obligations of national governments in the face of food and nutrition insecurity.

## Introduction

Food insecurity—defined as a “situation that exists when people lack secure access to sufficient amounts of safe and nutritious food for normal growth and development and an active and healthy life” [1]—sits firmly at the intersection of the rights to food and health, creating intersectoral opportunities for the implementation of rights-based legislation, policies, and programs for the realization of food security. Since the early 1990s, there has been a decline in food insecurity, undernutrition, and undernourishment [1]; however, food and nutrition security is expected to increase as a result of new challenges. Given expected rise in food insecurity, a response to these challenges requires a sufficient focus on a human rights-based approach to food security, including *nutrition* security.

Food security and nutrition security are interlinked and must be addressed simultaneously to address associated health challenges. Complex and multidimensional, food security is not limited to ensuring the sufficient production of food; it encompasses the need to guaranteed access and availability of nutritious food, a characteristic that has been stressed by international organizations, such as the World Health Organization (WHO), World Food Programme (WFP), as well as the Food and Agriculture Organization of the United Nations (FAO). FAO, for example, has emphasized that “[g]ood nutrition is the foundation for human health and well-being” and “physical and cognitive development” [2]. It helps to better protect human beings from disease. With public health linking nutrition to food, food and nutrition security must be tackled simultaneously to produce improved population health outcomes.

This article reviews the birth of a human right to food and nutrition security as a basis for public health, addressing its development under international law, its implementation in national policy, and its accountability through treaty bodies. Through this focus on the development and implementation of a human right to food and nutrition security, the article concludes by emphasizing the importance of food and nutrition security to human dignity, the very foundation of human rights, and the opportunities that a human rights-based approach offers to improve the lives of vulnerable populations in a rapidly changing world.

## Discussion

This section outlines the connections between food and nutrition security and public health, focusing on two of the key populations disproportionately affected by both food and nutrition insecurity: women and children. The focus on women and children is intended to highlight some of the major challenges with food and nutrition insecurity and not to undermine the importance of addressing the needs of other similarly affected populations like the aging, indigenous people, refugees, and internally displaced persons.

Having set this foundation, the article then reviews the parallel evolution of a human right to food and a human right to health under international law, highlighting the normative justification for framing food and nutrition security as an independent, intersectional human right.

## Food and nutrition security as an underlying determinant of health

Reflecting a growing understanding of its multidimensional character, the definition of food security has undergone many shifts over the years, moving from a food production-focused definition to one that largely embraces nutrition. The original definition arose in 1974 at the World Food Conference to focus on the “availability at all times of adequate world food supplies of basic foodstuffs to sustain a steady expansion of food consumption and to offset fluctuations in production and prices” [3]. Expanded over the ensuing years to focus on public health, the 1996 World Food Summit focused on the need for nutrition as a basis of health: “when all people, at all times, have physical and economic access to sufficient, safe and nutritious food to meet their dietary needs and food preferences for an active and healthy life” [4]. Today, the FAO conceptualizes food security as having four dimensions that should be fulfilled simultaneously: the physical *availability* of food, the economic and physical *access* to food, the body’s *utilization* of the nutrients found in food, and the *stability* of the previous three dimensions over time [5].

The focus on the body’s *utilization* of food for health essentially integrates nutrition security into food security, with the FAO defining nutrition security as “[a] situation that exists when secure access to an appropriately nutritious diet is coupled with a sanitary environment, adequate health services and care, in order to ensure a healthy and active life for all household members” [1]. By examining food, environment, and care together, nutrition security ceases to exist in the absence of food security, thereby more explicitly linking food security and health

The definition of “food security” has also come to include food safety, which is now recognized as key link between food and health [6]. Food safety requires all actions geared toward ensuring that food is as safe as possible [7]. In fact, in an effort to emphasize food safety as a critical component of food security, the FAO added the terms “safe and nutritious” to the definition of food security during the 1996 World Food Summit [8]. It is here where food security becomes strongly linked to water, sanitation, and hygiene (WASH). Lack of access to safe drinking water, sanitation, and hygiene can lead to infectious diseases like diarrhea and other intestinal diseases that can significantly undermine a person’s ability to absorb the necessary nutrients [9].

The FAO defines malnutrition as “an abnormal physiological condition caused by inadequate, unbalanced or excessive consumption of the macronutrients that provide dietary energy (carbohydrates, protein and fats) and the micronutrients (vitamins and minerals) that are essential for physical and cognitive growth and development” [2]. Malnutrition stands as a serious challenge to achieving both nutrition security and food security. Contrary to common misconception, malnutrition is not simply undernourishment. In fact, malnutrition is complex and can take multiple forms, all of which are tied to inappropriate nutritional diet. While undernourishment refers to the insufficient *intake* of food, undernutrition should be understood as its *outcome*, where a person’s body mass index (BMI) is equal to or falls below 18.5. While it is estimated that undernutrition has reduced globally—from 18.6% in 1990–1992 to 10.9% in 2014–2016—there remain 795 million who are undernourished [1] and approximately 2 billion with one or more micronutrient deficiencies (MNDs), especially iodine, iron, vitamin A, and zinc [10, 11]. Impacting public health, experts have underscored that MNDs “significantly contribute” to chronic diseases [12] and infectious diseases [13] in affected countries. The World Health Organization (WHO) has emphasized that “micronutrient malnutrition contributes substantially to the global burden of disease” and accounts for the “wide range of non-specific physiological impairments, leading to reduced resistance to infections, metabolic disorders, and delayed or impaired physical and psychomotor development” [12].

Furthermore, malnutrition affects one third of the world’s population, and close to half face more than one type of malnutrition [14]. It constitutes a serious economic development challenge for countries, especially for those that face a high prevalence in more than one type of malnutrition, including undernutrition and overweight. Facing this double burden of disease, there are over 1.4 billion people overweight across the world, including 500 million obese [2], with some nations’ overweight and obesity prevalence surpassing undernutrition [15, 16].

While malnutrition can affect anyone in any country, its harms fall most heavily on children and women of reproductive age, especially those in developing countries [11]. The global prevalence of malnutrition remains dire as a determinant of the health of the child, for whom long-term undernutrition and micronutrient deficiency can lead to stunting,^1^ [1] and in turn impaired cognitive and physical development [16]. Globally, 26% of children under five are stunted, with approximately 160 million in developing countries [2]. Additionally, with the globalization of the high-sugar and high-fat diets, which are conducive to overweight and obesity, malnutrition can mean not only undernourishment and undernutrition but also overnutrition of children [2]. Largely due to marketing schemes and lack of adequate regulation of the food industry, an issue that will be elaborated further below, childhood obesity has been on the rise, with an attendant rise in non-communicable diseases (NCDs), and there are now more overweight or obese children living in low- and middle-income countries than high-income countries. Between 1990 and 2014, Africa experienced a doubling in the number of overweight children under the age of five, from 5.4 million to 10.3 million. According to the WHO’s Commission on Ending Childhood Obesity, approximately 41 million children under 5 years of age were overweight or obese in 2014—25% in Africa and 48% in Asia [17].

Women are likewise significantly affected and should form an essential part of any intervention designed to address food and nutrition insecurity. In many societies, women continue to face serious obstacles as a result of deep-seated gender discrimination that interfere with household food security and nutrition [18]. As a result, women experience difficulty accessing, and controlling, resources, despite being key players in food production in many developing countries. In agricultural communities, it is women who not only care for the children but also work the fields, especially in societies where urbanization is on the rise and men in rural communities are forced to migrate into the cities for work. However, they lack access to land, credit, education, and agricultural inputs [19]. The high nutrition requirements during pregnancy and lactation coupled with gender-based discrimination present in many parts of the world make women (especially poor women) more prone to malnutrition than men [20]. In Southern Asia and sub-Saharan Africa, women are more likely to experience obesity and other nutrition-related chronic diseases than men [20]. In Vietnam, where two sets of household surveys conducted 5 years apart revealed that men reaped proportionally more nutritional benefit from economic development than women, as reflected in food access and body weight data [21].

Contributing to the health of the entire population, maternal nutrition is critical to tackling malnutrition among children. Maternal undernutrition leads to restricted fetal growth, infant deaths, and if the child survives, stunting [16]. As the FAO has emphasized, poverty promotes a vicious cycle of undernutrition: “Poverty often begins with poor nutrition and health, especially in early childhood.... Moreover, stunted girls grow up to become stunted mothers; maternal stunting is one of the strongest predictors of giving birth to a low-birth-weight infant. Maternal and child malnutrition thus perpetuate the cycle of poverty” [22]. Further, maternal overweight and obesity can increase the risk of the child becoming obese and developing non-communicable diseases later in life [16]. Therefore, laws and policies that limit women’s access to key resources—including health care, land, and credit—have a disproportionate effect on women’s food and nutrition security [18].

Considering these challenges, raising the nutritional quality of food to address micronutrient malnutrition has entailed food fortification (addition of micronutrients to processed foods), dietary diversification, and supplementation efforts for groups at risk, including children and women [11]. Alongside nutrition education and public health and food safety measures, food fortification policy has “proven effective for prevention of specific diseases, including birth defects” [23] and has been more strongly promoted by WHO since the 1990s. With the aim of “eliminating iodine deficiency disorders as a *major public health problem* in all countries by the year 2000” (emphasis added), the World Health Assembly in 1990 passed what has been deemed a “landmark resolution” for addressing micronutrient malnutrition, urging WHO Member States “to continue to give priority to the prevention and control of iodine deficiency disorders through appropriate nutrition programmes as part of primary health care” [24]. Since then, the international community has achieved greater consensus around food fortification,^2^ and more than 80 countries now have fortification policies to promote food and nutrition security [25].

## Global drivers of food and nutrition insecurity

The enjoyment of food and nutrition security at the community level is inevitably determined by factors taking place globally, such as climate change, increased urbanization, emergencies (both natural and human-made), and the globalization of unhealthy diet through the growth of the food industry. These global drivers of food and nutrition security call for innovating solutions involving efforts at the international level, and because they are more likely to affect vulnerable populations, a human rights approach is critical to comprehensively address their needs.

### Climate change

Climate change is expected to bring about increased hunger and undernutrition, exacerbating vulnerability to food and nutrition insecurity created by social, economic, and political processes [26]. Especially in developing countries that are already being significantly impacted by climate change, there is serious concern over the effects that it will have on the poorest and the most vulnerable—an observation that was raised during the 1990s and that continues to be emphasized by experts and the international community [27]. Population growth and demographic changes, whether triggered by climate change or other factors (e.g., violence and urbanization), also place a tremendous strain on how food is produced and distributed that together with climate change create serious challenges in ensuring food security of people worldwide.

Experts have observed that the magnitude or frequency of the climate-related event does not predetermine a climate change-related disaster, but rather, it is the population’s level of vulnerability and natural environment that will be a major determining factor [27]. In 2010, the UN System Standing Committee on Nutrition published a paper on climate change and nutrition that underscored the fact that vulnerable populations, including women and children living in marginal communities, are “at greatest risk to suffer from the potential impacts of climate change” [28]. Climate-related events, such as droughts, oftentimes lead families to adopt “negative coping strategies,” such as “reduction of the quality, safety, and quantity of their meals,” thereby increasing their “risk of undernutrition,” especially that of young children and women [28]. In response to this discourse, there has been a special emphasis placed on “vulnerability reduction in disaster management” so that affected populations can better “build resilience to rare, extreme, and potentially devastating climate events” [27]. These efforts would entail building greater food security and reducing the affected populations’ vulnerability to climate change.

Where climate change was long framed politically as an environmental issue [29], neglecting to emphasize the potential repercussions on global health, scientific discourse in the early 2000s began to link climate change to development, and in the last decade, health and human rights have increasingly become a major focus of the discourse on climate change mitigation and adaptation. These efforts would entail building greater food and nutrition security and reducing the affected populations’ vulnerability to climate change. Although governments initially focused on mitigating the effects of climate change under the 1992 United Nations Framework Convention on Climate Change (FCCC)—the first international treaty on climate change—links have been drawn between climate change and public health, and countries have recognized the co-benefits of voluntary mitigation actions to health, and emphasized the importance of a human rights-based approach to climate change. Starting in the UN’s human rights system, the UN Human Rights Council issued a 2008 resolution that drew attention to the human rights implications of climate change on sustainable development, particularly recognizing “that the world’s poor are especially vulnerable to the effects of climate change, in particular those concentrated in high-risk areas, and also tend to have more limited adaptation capacities” [30]. By the 2015 Paris Agreement, states would come together to call for efforts to “respect, promote and consider their respective obligations on…the right to health” as they undertake actions to address climate change [31].

The Paris Agreement, recognizing that the current situation urgently demands resiliency [27], calls for greater attention to adaptation strategies to fight food insecurity in order to minimize the impact of climate change in developing countries [32]. Drawing on the UN Standing Committee on Nutrition, which emphasized that “[p]lacing people and human rights at the center of strategies to adapt to and diminish the effects of climate change can enhance the development and implementation of climate-resilient policies,” [28] focus has shifted to reducing social factors that contribute to a population’s high vulnerability to food shortage within the context of climate change [27]. Moreover, disaster relief efforts responding to climate events should focus on addressing vulnerabilities rather than on “returning systems to previous conditions” [27].

### Urbanization

In 2014, the UN projected that the global urban population would grow by more than a 2.5 billion people by 2025—and that the rural population would stay the same. Ninety percent of that growth is expected to take place in Asia and Africa [33]. Increased urbanization, mostly a result of rural to urban migration, poses a serious threat to food security, particularly for the urban poor. This is especially true for those living in low-income countries, where the urban population is rapidly growing [34].

In these settings, food may be physically available, but what becomes the real obstacle is its affordability. The high cost of living in a city has been seen to undermine the urban poor’s ability to afford food and meet their basic nutritional needs, as they also look to cover their other essential needs, such as health care, transportation, and education [35]. The urban poor spend a large percentage of their income in food, and increases in food prices can leave this population highly vulnerable. According to study of 20 low- and middle-income countries, food expenditure among the extremely poor urban households ranged from 48% (Guatemala) to 74% (Tajikistan) [35].

Nutrition security in urban areas is highly dependent on its interplay with food, health and hygiene, and care [35]. For example, because mothers are more likely to work outside of their home in urban settings, there is a greater chance that their children will receive less nutrients and immunity to disease as a result of mothers reducing the breastfeeding period by 2 to 3 months. Furthermore, many countries experiencing rapid economic growth are faced with changes in demographics that outpace their ability to make the necessary political and social reform, including ensuring food and nutritional security for the most vulnerable populations [36].

### Emergencies

Natural and human-made disasters, including those related to climate change and social conflict, can lead to disruptions in food production, physical access to food, and food safety efforts especially where and when health care systems and sanitation are compromised. Over the last three decades, we have observed not only a rise in armed conflict but also an increase in their duration [1, 37]. As a result, food and nutrition insecurity among the affected populations, particularly refugees and internally displaced persons (IDPs), becomes especially problematic. For instance, between 2010 and 2012, 250,000 people died in Sudan from the famine following armed conflict and a drought—many more than the number of people who died as a direct result of the conflict [1]. In Syria, the start of 2015 saw 9.8 million people needing “various levels of food, agriculture, and livelihood-related assistance” and 6.8 million of them were in “critical need of food assistance” [1].

With women and children being the target of armed conflict, their vulnerability is exacerbated, and that includes their food and nutrition insecurity. A 1994 study of Kurdish refugees in Iraq demonstrated that children under two experienced “significant weight loss” within the first month of the conflict and “the prevalence of acute malnutrition was threefold the normal range in children aged 12 to 24 months” [37, 38]. In 2002, the U.N. Secretary General reported to the Security Council that “[w]here cultures of violence and discrimination against women and girls exist prior to conflict, they will be exacerbated during conflict” [39]. Additionally, where women and girls are food producers, they are faced with the responsibility of ensuring food security for their household as males in the family participate in the armed conflict while lacking land and property rights and control of resources to do so [39].

### Growth of the food industry and the globalization of an unhealthy diet

Through the lifting of trade barriers and inadequate domestic regulation, globalization has meant the growth of the food industry and the rise of an industrial epidemic that threatens global nutrition security. Multinational companies of ultra-processed foods and sugar-sweetened beverages either reached or expanded their reach among populations in low- and middle-income countries. Marketing schemes, coupled with inadequate regulation at the domestic level, have shifted patterns of consumption in many countries. It is estimated that processed foods account for 75% of food sales worldwide, and more than one third of this global market is controlled by their major manufacturers [40]. Consequently, we have observed the globalization of unhealthy diet and a rise in the prevalence of non-communicable diseases (NCDs) globally—disproportionately impacting low- and middle-income countries.

NCDs, such as cardiovascular disease and diabetes, account for 38 million [41] or 70% of deaths worldwide [42], making NCDs the leading cause of death globally. Twenty-eight million, or three-quarters, of the total deaths take place in low- and middle-income countries, and 82% of the 16 million NCD-related “premature deaths” (before the age of 70) worldwide occur in these countries [41]. Unhealthy diet, which accounts for 2.7 million deaths annually, constitutes a major risk factor for NCDs [43].

There is growing evidence that regulation strategies, such as taxes and labeling restrictions, can be effective in combatting the disease epidemic. For example, in January 2014, Mexico imposed a 10% tax on sugary drinks in response to the high prevalence of diabetes and obesity in its population, resulting in a 12% drop in sales within the year [44]. In Mauritius, regulation measures to reduce saturated fatty acids in cooking oil by replacing it with soybean oil has improved consumption patterns and average total cholesterol levels [45].

There is also greater awareness about the contribution of major food companies to this industry epidemic, which has increased efforts to regulate their products and activities, including marketing and product labeling. As elaborated in the next section, state obligations that arise from a human rights-based approach can support and promote measures to better control and guide industry activities and help to reverse consumption patterns, thereby increasing nutrition security.

Human rights provide a path to address these challenges, averting drastic social changes on a population critically affected through interconnected obligations to realize the right to food and the right to health. Therefore, a human rights-based approach should inform laws and policies developed, especially with regards to the needs of the most affected populations like women and children [46].

## Human rights as a tool to address food and nutrition insecurity

Human rights offer universal frameworks to advance global justice for food and nutrition security. Instrumental to human dignity, rights seek to address basic needs and frame individual entitlements to uphold a universal moral vision [47]. By addressing threats as “rights violations,” international law offers global standards by which to frame government responsibilities and evaluate policies and outcomes under law, shifting the policy debate from political aspiration to legal obligation [48]. Empowering individuals to seek accountability for these government obligations rather than serving as passive recipients of government benevolence, human rights law identifies individual rights-holders and their entitlements and corresponding duty-bearers and their obligations [49]. The state becomes the duty bearer under international law upon ratification of the underlying human rights treaty, with the government thereafter accepting resource-dependent obligations to “progressively realize” rights “to the maximum of its available resources, with a view to achieving progressively the full realization of the rights” [50, 51].

The codification of human rights under international law begins in the aftermath of World War II, with rights related to food and health serving to prevent deprivations like those that had taken place during the depression and the war that followed. [52] As a basis for building a just world out of the ashes of war, states worked under the auspices of the nascent UN General Assembly to enumerate and elaborate these human rights under international law, proclaiming on December 10, 1948, a Universal Declaration of Human Rights (UDHR) to create “a common standard of achievement for all peoples and all nations” [53]. With the UDHR developing a right to “a standard of living adequate for the health and well-being,” it specifically clarified a series of underlying determinants of health, “including food, clothing, housing and medical care and necessary social services, and the right to security in the event of unemployment, sickness, disability, widowhood, old age or other lack of livelihood in circumstances beyond his control” [53]. Building from this non-binding declaration, states continued to negotiate in the ensuing years to develop specific legal obligations under two separate human rights covenants, enacting in 1966 the International Covenant on Civil and Political Rights (ICCPR) and the International Covenant on Economic, Social and Cultural Rights (ICESCR). These three documents—the UDHR, ICCPR, and ICESCR, adopted separately by the UN General Assembly and referred to collectively as the “International Bill of Human Rights”—form the normative basis of the human rights system from which the human right to food and the human right to health would evolve as interconnected rights under international law.

The human rights to food and health have evolved dramatically over the past 70 years to encompass food and nutrition security, with international law reflecting the negotiated codification of global norms and reifying those norms until revised through normative evolution and subsequent legislative or jurisprudential amendment [54]. Despite the political constraints of the Cold War, states sought to formalize these rights in the 1966 ICESCR, providing under the following:

- Article 11—“the right of everyone to an adequate standard of living for himself and his family, including adequate food, clothing and housing, and to the continuous improvement of living conditions,” with states “recognizing the fundamental right of everyone to be free from hunger” and including specific obligations “to ensure an equitable distribution of world food supplies in relation to need.”
- Article 12—“the right of everyone to the enjoyment of the highest attainable standard of physical and mental health,” including specific obligations on states to take all steps “necessary for the improvement of all aspects of environmental and industrial hygiene” and for “the prevention, treatment and control of epidemic, endemic, occupational and other diseases” [50].

Drawing on these seminal codifications of rights-based obligations, the UN continued to advance efforts to address hunger and malnutrition as a harm to public health [55], with food and nutrition security obligations developed to address the specific human rights of:

- Women—with the 1979 Convention on the Elimination of All Forms of Discrimination Against Women finding a woman’s right to health to include “adequate nutrition during pregnancy and lactation;” [56] and
- Children—with the 1989 Convention on the Rights of the Child codifying a government obligation “to combat disease and malnutrition, including within the framework of primary health care, through, *inter alia*, the application of readily available technology and through the provision of adequate nutritious foods…” [57]

As the Cold War came to an end, there arose a renewed international commitment to human rights, with international recognition in the 1990s that “[a]ll human rights are universal, indivisible and interdependent and interrelated” [58] creating a political space to address food security as central to individual dignity [4]. Shifting from global food supply to household food security, human rights scholarship at the intersection of food and health began to transition from a focus on calorie counts to an analysis of nutritional content [59]. Building from the UN’s drafting of the 2000 Millennium Development Goals—which focus pervasively on health and begin with a focus on the eradication of hunger—these human rights interpretations would seek to translate global norms into state obligations.

Memorializing such interconnected human rights and corresponding government duties, the UN Committee on Economic, Social and Cultural Rights (CESCR, the legal body charged with drafting official interpretations of, and monitoring state compliance with, the ICESCR [60]) issued a series of General Comments to provide authoritative interpretation of the norms inherent in these rights: in 1999 on the right to adequate food [61] and in 2000 on the human right to health [62].

In focusing on the right to food, General Comment 12 found under article 11 of the ICESCR:

- The right to adequate food is realized when every man, woman and child, alone or in community with others, have physical and economic access at all times to adequate food or means for its procurement [61].
- Focused on the “adequacy” of food, the CESCR connected food to health in a way that implicates a diet containing “a mix of nutrients for physical and mental growth, development and maintenance, and physical activity,” concluding that “[e]very State is obliged to ensure for everyone under its jurisdiction access to the minimum essential food which is sufficient, nutritionally adequate and safe, to ensure their freedom from hunger” [61].

To reflect a modernized right to health commensurate with an understanding of determinants of health, General Comment 14 interpreted article 12 of the ICESCR to find that:

*the right to health embraces a wide range of socio-economic factors that promote conditions in which people can lead a healthy life, and extends to the underlying determinants of health*, *such as food and nutrition*, housing, access to safe and potable water and adequate sanitation, safe and healthy working conditions, *and a healthy environment*. (emphasis added) [62].

The CESCR thereby included “an adequate supply of food and proper nutrition” under the right to health, linking the quantity and quality of food necessary for a healthy diet and thereby articulating a core state obligation “[t]o ensure access to the minimum essential food which is nutritionally adequate and safe, to ensure freedom from hunger to everyone” [62].

With these general comments sharing an interconnected focus on nutrition, the CESCR finds the right to food and the right to health to be intersectional rights—interdependent and interrelated and encompassing an array of underlying factors that impact health, with specific state obligations to address food insecurity as a means to adequate nutrition. As other human rights treaty bodies have come to address the specific health rights of women and children, they too have come to focus on the interconnections between food and health, with general comments and recommendations that elucidate the health harms of inadequate food supply [63] and the state obligations for adequate nutritious foods (with a malnutrition focus on both undernutrition and overnutrition) [64].

With the UN Commission on Human Rights developing a Special Procedure mandate for a Special Rapporteur on the Right to Food in 2000 [65] and a Special Rapporteur on the Right to Health in 2002 [66], these special rapporteurs have sought to clarify their respective rights while recognizing, if not addressing, their intersectionality. The Special Rapporteurs on the Right to Health have advanced an availability, accessibility, acceptability, and quality framework to elaborate the norms of the right to health, with annual UN reports on the health implications of hunger, the links between unhealthy foods and non-communicable diseases, and the role of nutrition in early childhood development [67, 68]. The Special Rapporteur on the Right to Food has similarly advanced an availability, accessibility, adequacy, and sustainability framework for the right to food, defining this right to provide “physical and economic access at all times to sufficient, adequate, and culturally acceptable food that is produced and consumed sustainably, preserving access to food for future generations” and applying this right to focus holistically on the health harms of malnutrition (including undernutrition, micronutrient deficiency, and overnutrition) [69, 70]. While clarifying their respective rights, however, the right-specific mandates of these special rapporteurs have limited them in addressing areas where these rights intersect.

Conceptualizing an intersectional right to food and nutrition security, reflecting both the evolving definitions of food security and the evolving codification of food and health rights, would encompass the rights of households to adequate nutrient content through sustainable foods. Where scholars have long seen “the validity and the necessity of a dynamic approach to human rights,” they have cautioned that the proliferation of new rights is often developed in a “haphazard, almost anarchic manner” [54]. At the intersection of the right to food and the right to health, this collective right to food and nutrition security moves beyond the food necessary for survival and encompasses the nutrition necessary for health [71]. An understanding of food security has developed alongside an increased focus on nutrition as a basis for health, yet in translating this food security understanding into human rights obligations, neither the CESCR’s General Comments 12 on the right to food nor the CESCR’s General Comment 14 on the right to health has fully addressed these normative concerns or fully developed accountability mechanisms to assure their realization [72]. Where paradigms of food and nutrition security are not reflected in current discourse, a new human right is warranted to reflect evolving understanding of contemporary threats to human dignity and well-being. General Comments 12 and 14 are an initial, though incomplete, part of this evolving notion of human rights for food and nutrition security. Addressing two rights at their intersection—similar to the approach taken by the CESCR in General Comment 15 in developing a right to water out the right to health and the right to an adequate standard of living—provides a basis to develop a new right reflective of the interconnections across underlying determinants of health. Such an intersectional right encompasses normative pillars for:

- Availability (National Level)—Facilitating stability and sustainability of the national food supply, the availability of food security implicates both national agricultural production and global food markets. In a globalized food market, such collective availability concerns demand attention to global determinants of food security, including economic development policies, market systems, and sustainable agriculture [73].
- Accessibility (Household Level)—For states to adjust to changes in increasingly integrated global food markets, it is necessary that food remain geographically and financially accessible across households, without discrimination in the distribution of food within households [61]. Rather than focusing on global or national deprivations, the level of focus on food security implementation should be at the household level, addressing “access at the site where food is consumed to other processes and factors that relate access to intra-household distribution and individual dietary intake” [59]. This focus on intra-household distribution highlights the importance of equity in allocations of food.
- Acceptability (Within Household Level)—Equitable utilization of food requires attention to food acceptability, focusing on culturally appropriate foods for necessary health outcomes and acknowledging the particularized nutrition needs for the health of vulnerable populations (including among pregnant and nursing women, children, and the elderly).
- Quality/Adequacy (Individual Level)—Moving beyond quantity to address the quality of food, the concept of “adequacy” addresses nutrition as an underlying determinant of health [74]. This focus on nutrition through attention to the adequacy of food links a right to food and nutrition security to both the right to food and the right to health. As an intersectional right that implicates food as a basis for health, it is necessary to look beyond a mere right to subsistence through a minimum number of calories and examine the larger issues of quality through nutritional standards [75].

This intersectional conceptualization brings into focus underlying determinants of health resulting from food and nutrition security: providing proper nutrition for physical and mental development and the prevention of diseases caused or abetted by malnutrition—either undernutrition, nutrient deficiency, or overnutrition. As a central component of human dignity, food and nutrition security can empower households to look beyond the uncertainty of hunger and malnutrition through government obligations to secure the conditions for a healthy, energized life.

From human rights to state obligations, the realization of a right to food for the protection of food and nutrition security under international law necessitates intersectional national policies. Food and nutrition security is assured through food sovereignty, understood as “the right of peoples to healthy and culturally appropriate food produced through ecologically sound and sustainable methods, and their right to define their own food and agriculture systems” [72]. By upholding the rights of producers, food sovereignty serves as a precondition for the right to food [76] and provides a basis by which states can be resilient in adjusting to global disruptions in food production and distribution. Such a shift toward national control over food systems implicates the need to translate the development of a right to food and nutrition security under international law into the implementation of this intersectional right through national policy [77].

## Recommendations

Recognizing the limitations of international law in affecting the practice of national governments, this section explores and recommends avenues through which human rights obligations could be translated into national laws, policies, and programs, thereby operationalizing international law at the domestic level to address food and nutrition insecurity. Facilitating international accountability for national practice, the UN’s human rights treaty bodies provide space for civil society advocacy and constructive state dialog on the effective implementation of international human rights obligations.

## Domesticating international law through national policy

The development of a human right to food and nutrition security under international law has codified an imperative to implement human rights in food insecurity policy. Where international law has no direct (or self-executing) effect on national governments, it becomes necessary to operationalize state human rights obligations through national laws, policies, and programs. [78] With policymakers, practitioners, and advocates translating international human rights into rights-based outcomes, these stakeholders implement rights through national policy [79], invoking a rights-based approach to food and nutrition security policy as a means to: frame the legal and policy environment, integrate core principles into policy and programming, and facilitate accountability for obligations [80]. Where food rights have long been thought of as “vague, if not unclear,” [81] the domestic codification of a right to food and nutrition security could concretize this right through its application [75]. Translating international law into national policy and practice, human rights are seen to “cascade” down to the national level, by which these norms gain broader international acceptance through national policy, and state duty-bearers internalize obligations to progressively realize rights through food insecurity policy [82].

Seeking to implement international law at the national level, the UN’s specialized agencies have provided support to states in implementing rights to food and nutrition security. The FAO worked with an Intergovernmental Working Group to develop the “Voluntary Guidelines to support the progressive realization of the right to adequate food in the context of national food security,” which provide practical guidance to states in their implementation of the progressive realization of the right to adequate food in the context of national food security [73]. In focusing states on the link between adequate food and disease prevention, Guideline 10 (Nutrition) encourages states to address food security through healthy eating habits, thereby preventing malnutrition, overconsumption, and unbalanced diets [73]. Supporting these efforts to realize food and nutrition security, the FAO’s Voluntary Guidelines encourage states to pursue food security through, inter alia, inclusive land-use policies (particularly expanding land access for women), social safety nets (to assure economic accessibility), local and regional markets (through transportation, storage, and distribution infrastructure), and sustainable agriculture (supporting farmers to diversify their crops based on nutritional content) [73]. Working with WHO and Office of the United Nations High Commissioner for Human Rights (OHCHR), FAO has followed up this governmental guidance with programmatic guidance to promote nutrition, recognizing the role of nutrition as an underlying determinant of health [74]. The WFP Nutrition Policy approved in 2012 includes previous policies on nutrition topics and strategies through which the WFP engages in nutrition, outlining nutrition-specific and nutrition-sensitive programs [83].

The range of international legal and practical guidance instruments available at the international level provide states with a basis on which to build a human rights-based and sound implementation approach to addressing food and nutrition insecurity at the national level. Recently, the World Health Assembly issued guidance on the importance of abiding by international obligations, as Member States look to undertake measures to combat the inappropriate marketing of foods. It urged them “to take all necessary measures in the interest of public health to end the inappropriate promotion of foods for infants and young children, including, in particular, implementation of the guidance recommendations while taking into account existing legislation and policies, as well as international obligations” [84].

Domestication of international law can be accomplished through national constitutions, courts, and legislation.

- National Constitutions—National constitutions are important vehicles for formally recognizing the rights to food and health at the domestic level. Constitutions may either recognize these rights explicitly or may refer to food, food security, or nutrition as “directive principles,” or statements of principles that are intended to guide government action. There are 23 national constitutions worldwide that recognize the right to food [85] and close to half of the world’s constitutions enshrine the right to health [86, 87]. The Constitution of Ecuador, a country experiencing high level of food insecurity as a result of varying social economic factors [88], explicitly recognizes the right to food under Article 13, which states: “[t]he Right to Food includes the free and permanent access to sufficient innocuous and nourishing food for a healthy and quality feeding, in accordance with the culture, traditions and customs of the peoples. The Ecuadorian State will recognize and guarantee the right to food sovereignty.” In the case of Bangladesh, which faces high levels of undernutrition as a result of natural disasters [89], the national constitution refers to food within directive principles. Article 15 (Provision of Basic Necessities) recognizes both food and medical care as basic necessities of life. Article 18 (Public health and morality) establishes “the raising of the level of nutrition and the improvement of public health as [the State’s] primary duties” [90]. Thus, constitutions are keys to not only raising food security to an individual entitlement but also expanding its definition as such. Such constitutional provisions create the necessary foundation for the development or reform of national policies and legislation for the promotion of food and nutrition security.
- National Legislation—National legislation is critical to domestically implementing international law, especially where treaties are not automatically incorporated into domestic law and require specific legislative action to given treaty provisions legally binding effect. Accordingly, human rights-based protection of food and nutrition security requires that states harmonize their national laws in accordance with international standards and their own domestic context. In this manner, implementing legislation serves as the bedrock from which strong policies and the necessary programs to improve food and nutrition security can be developed. In fact, in interpreting the right to food, the CESCR has explicitly stated that “implementation at the national level” requires the adoption of “framework legislation” as a “major instrument” of the country’s national strategy, where the scope and the content of the right, overarching principles, obligations of relevant actors, and the necessary implementing mechanisms are established [61, 91]. It has further emphasized that UN specialized programs and agencies, such as the FAO and United Nations Children’s Fund (UNICEF), are well equipped to provide states with support in drafting framework legislation [61]. It is worth noting that the use of innovative approaches, such as social epidemiological profiles, can help support the application of human rights to domestic legislation in a more practical manner by going beyond the traditional age, sex, or socio-economic status and capturing the related health factors or demographic characteristics that more accurately reveal the unmet needs of populations affected by food insecurity [92].
- Litigation—Litigation can serve as an important avenue for the vindication of human rights and as a mechanism through which the justiciability of the right to food is realized and can be claimed individually and collectively. While not as expansive as that of the right to health, right to food jurisprudence has been developed. The Indian Supreme Court, in particular, has not been shy about acknowledging the existence of right to food in its case law despite the lack of an explicit constitutional protection of the right to food [93–95]. In the 1989 case of *Pattnayak & Another v. State of Orissa*, the Court recognized the right to food as part of the right to life, enshrined under Article 21 of the Indian Constitution. Moreover, as underscored by the FAO, grounded on this established interrelationship, interim orders issued in *People’s Union for Civil Liberties (PUCL) v. Union of India and others* “led to new and better-implemented government programmes and … asserted that benefits under these programmes are legal entitlements,” which included “mid-day meals for school children, food entitlements in childcare centres, subsidized food for a number of specific vulnerable groups, as well as changes to the subsidies directed at all persons below the official poverty line.” Where the right to food has not proven as justiciable at the right to health, however, a right to food and nutrition security can provide measurable indicators by which to assess national efforts to progressively realize human rights.

Hence, these national legal avenues are critical to driving the implementation of international standards forward, imploring duty bearers to take action as a result of legal action framed in the language of a justiciable right to food, and to allowing for improved responses to food and nutrition insecurity on the ground. However, as discussed in the next section, international human rights bodies provide accountability mechanisms that can trigger or strengthen governments’ efforts to address the issue.

## Facilitating international accountability for national policy

Focusing on food and nutrition security as an intersectional right poses obstacles to accountability for national implementation; however, international accountability mechanisms exist through the UN’s human rights treaty bodies. Human rights treaty bodies monitor state implementation of the international human rights treaties, facilitating accountability for rights realization through their formal review of state reports, constructive dialog with state delegations, and concluding observations on state obligations. Comprised of independent experts, who are elected in their individual capacity rather than as representatives of their states, treaty bodies have international legal authority to assess whether state parties are implementing their treaty obligations [96]. In addition to clarifying treaty provisions through general comments, recommendations, or statements [97], these treaty bodies also review state reports on the implementation of rights within their monitoring purview [98]. All core human rights treaties require that states report regularly on the steps they have taken to implement their duties [99] and the goal for these reports is not only to assess the implementation of rights but also to bring about the opportunity to debate these human rights within the country. Through the findings of these reports, the treaty body engages in constructive dialog with the state and issues concluding observations or recommendations. These treaty body authorities influence states and galvanize advocates to take action to realize rights, with subsequent state reports seen to respond to issues raised in previous concluding observations [100, 101].

At the intersection of the right to food and the right to health, the right to food and nutrition security has been addressed principally by the CESCR, the Committee on the Rights of the Child, and the Committee on the Elimination of All Forms of Discrimination Against Women. As seen in the CESCR, states are seen to report on food or nutrition security—with attention to the safety and nutritional adequacy of food (with focus on wasting, underweight, and stunting)—and the Committee has responded by recommending the adoption of “effective and urgent measures to combat hunger and malnutrition” [102]. Where state reports regularly discussed assessment of nutrition-related health indicators, fortification, and breastfeeding promotion—as assessing realization of a right to food through quantity of calories is easier to report and analyze than measuring the nutritional food content necessary for disease prevention and health promotion—states have begun to report on policies for the progressive realization of rights related to food and nutrition security, with states seen to report on the quality of food through: infrastructural improvements [103], food safety programs [104], healthy food policies [105, 106], and food support for women and young children [107]. Complemented by the 2008 ICESCR Optional Protocol, developing a supranational individual complaint mechanism under the CESCR, the role of treaty bodies will prove increasingly relevant to implementation and accountability for a right to food and nutrition security [108].

These binding obligations are reinforced by a growing architecture of monitoring mechanisms in the UN system. The Universal Periodic Reporting (UPR) process and the country missions of the special rapporteurs provide mechanisms to facilitate accountability for state implementation efforts to progressively realize a right to food and nutrition security. These efforts have been recently reinforced by the adoption of the 2030 Agenda for Sustainable Development. Paragraph 72 voices the UN General Assembly’s commitment to “a robust, voluntary, effective, participatory, and integrated follow-up and review framework” [109]. This commitment supports the monitoring infrastructure of a right to food and nutrition security by emphasizing cross-sectoral goals based on the rights to food and health, as well as a broader recognition of the role of human rights in accomplishing the Sustainable Development Goals (SDGs). Statements and resolutions by the Secretary-General [110] and the General Assembly [111] of the United Nations, as well as the presentation of the first 22 voluntary country reports in the SDG era [111], show continued international commitment to national monitoring.

Considering the wide range of international and national accountability mechanisms, it is important to underscore the importance of civil society in facilitating accountability for food and nutrition security and their role as partners providing expert technical capacity and financial support within countries. Civil society actors engage in a variety of activities at both the national and international levels that must not be overlooked: pressuring governments through advocacy efforts to comply with international human rights obligations, monitoring this compliance, investigating the human rights situation, providing data, and litigation. Within the international system, pursuant to the periodic reporting requirements described above, non-governmental organizations (NGOs) can also submit “shadow reports” to UN treaty bodies to either confirm or challenge reports submitted by governments. Additionally, NGOs are invited to “attend the UPR Working Group sessions and can make statements at the regular session of the Human Rights Council when the outcomes of the State reviews are considered” [112]. It is worth noting that the CESCR recognizes their importance to the whole process. For example, with respect to the right to food, the CESCR has held that establishing national legislation requires the “active involvement” of civil society. States are expected to specifically provide for collaboration with civil society, among other sectors, in its right to food framework legislation [61]. NGOs can thus play a critical role in furthering the protection of human rights and fostering accountability intersection obligations pursuant to the right to food and nutrition security.

## Conclusion

Food and nutrition security is central to individual dignity and foundational to the enjoyment of human rights. People’s ability to access food is heavily defined by structural and social conditions. As climate change exacerbates food and nutrition insecurity, negatively impacting the health of the most vulnerable, climate change adaptation strategies must address the rights to food and health [28], protecting the well-being of disproportionally affected populations by adopting a human rights-based approach to food and nutrition security. This approach opens a series of international legal mechanisms through which food and nutrition insecurity can be addressed. Both the nature of the issue and the human rights-based approach call for complex interventions that involve a wide range of actors, including governments, affected populations, civil society, human rights ombudspersons, academics, international organizations, and the private sector that can together work toward the effective domestication of international standards for promoting food and nutrition security and thus ensuring better health outcomes for those affected.

## Abbreviations

BMI: Body mass index
CESCR: Committee on Economic, Social and Cultural Rights
FAO: Food and Agriculture Organization of the United Nations
ICCPR: International Covenant on Civil and Political Rights
ICESCR: International Covenant on Economic, Social and Cultural Rights
MNDs: Micronutrient deficiencies
NCDs: Non-communicable diseases
NGOs: Non-governmental organizations
OHCHR: Office of the United Nations High Commissioner for Human Rights
PUCL: People’s Union for Civil Liberties
SDGs: Sustainable Development Goals
UDHR: Universal Declaration of Human Rights
UN: United Nations
UNICEF: United Nations Children’s Fund
UPR: Universal Periodic Reporting
WFP: World Food Programme
WHO: World Health Organization

## Footnotes

1 FAO defines “stunting” as “[l]ow height for age, reflecting a past episode or episodes of sustained undernutrition.”

2 Conferences around the issue have included the Montreal conference on Ending Hidden Hunger in 1991, the 1992 FAO/WHO International Conference on Nutrition held in Rome, the 1993 World Health Assembly held in Geneva, and the 2002 Special Session on Children of the United Nations General Assembly. Public–private coalitions have also been created, including the Global Alliance for Improved Nutrition and The Global Network for Sustained Elimination of Iodine Deficiency.
